# Synaptic transmission in supragranular layers of the human cortex – comparative review of structure, function, and plasticity

**DOI:** 10.3389/fnsyn.2025.1724377

**Published:** 2025-12-10

**Authors:** Amelie Eichler, Pia Kruse, Charlotte Schob, Maximilian Lenz

**Affiliations:** Institute of Neuroanatomy and Cell Biology, Hannover Medical School, Hannover, Germany

**Keywords:** synapse, human neocortex, synaptic plasticity, neurosurgical resections, human neuronal networks

## Abstract

Synapses are the highly specialized connection sites between neurons enabling the establishment of complex neuronal networks. As highly plastic structures, synapses collocate both the transmission and storage of information, which is an essential prerequisite for learning and memory. Since synaptic deficits are associated with degenerative and neuropsychiatric diseases, it is essential to understand the mechanisms of synaptic plasticity. Throughout evolution, the human brain has developed distinct characteristics, such as supragranular expansion and enhanced long-range connectivity, suggesting an evolutionary specialization of synapses. Recent collaborative research, employing slice preparations obtained from neurosurgical resections of the human neocortex, has significantly advanced our understanding of the unique structural and functional properties of the human neocortex. This review investigates findings derived from diverse experimental methodologies, highlighting specific synaptic features. Focusing on synapses in supragranular layers, we discuss the distinctive synaptic structure, function, and mechanisms of plasticity that contribute to the unique circuitry of the adult human brain. Additionally, we outline emerging directions of research aimed at further elucidating the functionality of human cortical networks.

## Introduction

Higher brain function relies on orchestrated interactions between neurons in complex networks. Synapses are specialized contact sites between neurons that collocate signal transmission and storage, which underlines their essential role in physiological network function. Therefore, synaptic transmission and plasticity, both on structural and functional levels, were intensely investigated over the last decades in various cell culture models and living animals. However, our knowledge on synaptic physiology in the human brain remains limited.

Recent work demonstrated a specialization of the human neocortex. The human neocortex, especially glutamatergic neurons in supragranular layers (layers 2/3), has expanded throughout evolution (Sousa et al., 2017; Berg et al., 2021), raising the question of how synaptic structure and function in these layers differ from those in commonly studied rodent models. Since excitatory synapses in the neocortex – predominantly glutamatergic contacts onto dendritic spines of pyramidal neurons – are essential for cortical information processing, understanding their physiology is crucial. Indeed, emerging evidence over the last two decades indicated that human layer 2/3 pyramidal neurons possess more extensive dendritic trees, unique membrane properties, and a greater number of excitatory synapses per cell compared to rodent neurons (Hunt et al., 2023; Mohan et al., 2015). These distinctions suggest that human cortical circuits may integrate information in a markedly different fashion. However, core principles of synaptic function seem to be conserved across species.

In this review, we focus on the structure, function, and plasticity of synapses in the human cortex and discuss how they resemble or diverge from *in vitro* and *in vivo* rodent models. After a general introduction to the basic concepts of synaptic transmission and plasticity, we will discuss current methods to assess synaptic transmission in the human brain. Subsequently, we will review current knowledge on synaptic commonalities and differences between humans and rodents.

## Synaptic transmission and plasticity

The presence of excitatory synapses is preserved across species, using glutamate as their major neurotransmitter. In principal neurons, glutamatergic synapses can often be associated with dendritic spines, which are membrane protrusions originating from the complex dendritic trees of neurons. Dendritic spines are plastic structures of variable size that link excitatory synaptic transmission zones onto the spine head to the dendrite via the spine neck. Thereby, synaptic signals are compartmentalized within dendritic spines before reaching the dendrite or soma of a neuron (Cornejo et al., 2022). However, numerous other classes of excitatory synapses exist that do not rely on the formation of dendritic spines, such as excitatory shaft synapses or synapses onto distinct interneurons.

**FIGURE 1:**
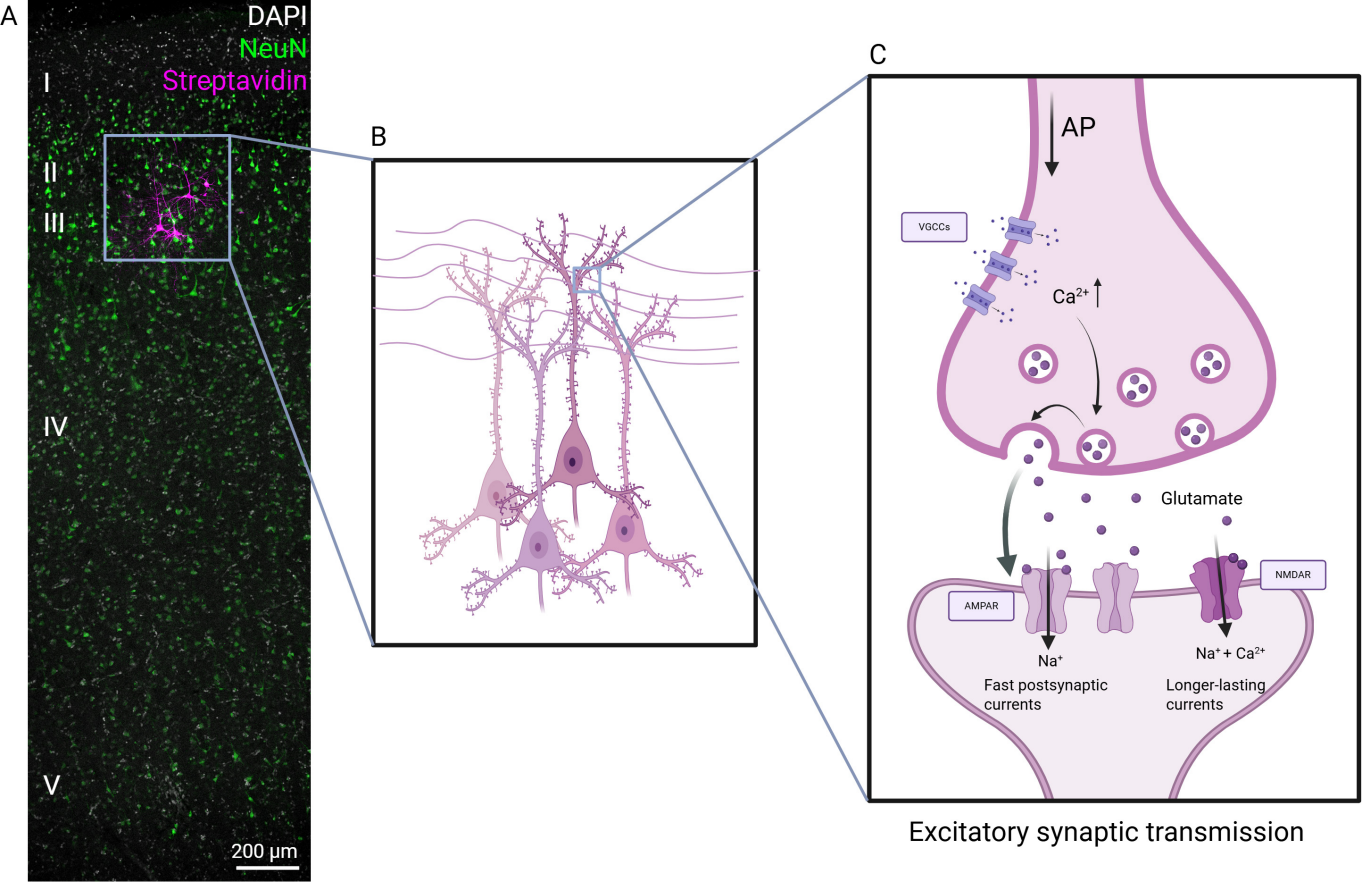
Excitatory synaptic transmission in the human neocortex. (A) Sample image of a human neocortical tissue section. Cytoarchitecture and lamination are indicated by DAPI and NeuN staining. Layer 2/3 (II/III) pyramidal neurons were filled with biocytin during electrophysiological assessment and are visualized by *post hoc* streptavidin staining. Scale bar, 200 μm. (B,C) Excitatory synaptic transmission of pyramidal neurons in the human neocortex. AP, action potential; VGCCs, voltage-gated calcium-channels. Created with Biorender.

Synapses are characterized by complex protein networks in both pre- and postsynaptic compartments that orchestrate fundamental processes, such as the presynaptic vesicle cycle or signal transduction at postsynaptic sites (Husi et al., 2000; Wang et al., 1997). In presynaptic boutons, incoming action potentials lead to a Ca^2+^ driven release of glutamate from synaptic vesicles attached to presynaptic active zones. Glutamate diffuses across the synaptic cleft and binds to postsynaptic receptors, predominantly AMPA- and NMDA-receptors (Figure 1). AMPA-receptors mediate fast excitatory postsynaptic currents (EPSCs) that depolarize the postsynaptic membrane, while NMDA-receptors produce slower, longer-lasting currents that are highly sensitive to coincident activity due to a Mg^2+^ block (Khazipov et al., 1995). Synaptic transmission is terminated by desensitization of postsynaptic receptors and clearance of neurotransmitters from the synaptic cleft through adjacent astrocytes (c.f., tripartite synapse). Although similar mechanisms can be found in rodents and humans, human synapses may have larger conductance per contact and specialized properties of signal integration as discussed below.

A defining feature of synapses is their collocation of transmission and information storage, which can be achieved by stimulus-driven changes in their structural and functional properties (Oh et al., 2015). Synaptic plasticity can operate on distinct time-scales, covering changes within milliseconds toward persistent long-lasting changes over years. Short-term plasticity (STP) dynamically modulates synaptic strength on the timescale of milliseconds to seconds, depending on recent history of synaptic activity and overall synaptic reliability (Vandael et al., 2021). In STP, repetitive presynaptic firing can lead to synaptic facilitation (transient increase in EPSC amplitude, often due to presynaptic Ca^2+^ accumulation) or synaptic depression (transient decrease in amplitude due to vesicle depletion or receptor desensitization). The balance of facilitation vs. depression is largely determined by release probability and vesicle dynamics (Taschenberger et al., 2016). Synapses onto different postsynaptic cell types exhibit characteristic short-term dynamics: while excitatory synapses onto certain inhibitory interneurons or long-range-projections often facilitate, synapses in recurrent connections often depress (Campagnola et al., 2022). As a result, the dynamics of individual unitary connections shape transmission by making synapses respond in a history-dependent manner, which is relevant for filtering sustained high-frequency activity or contrasting novel inputs.

Long-term synaptic plasticity alters synaptic efficacy for minutes to years and is thought to underlie learning and memory storage (Morris et al., 1986). Based on positive feedback loops between network activity and synaptic strength, long-term potentiation (LTP) leads to an activity-induced long-lasting increase in synaptic strength, long-term depression (LTD) causes a lasting decrease in strength. Classic rodent studies demonstrated that coincident pre- and postsynaptic activity, such as high-frequency stimulation or precise spike timing protocols, can trigger LTP or LTD at excitatory synapses via postsynaptic NMDA-receptor activation and the resultant Ca^2+^ influx (Malenka et al., 1988). Subsequently, CaMKII, calcineurin, PKA and other signaling molecules mediate synaptic plasticity through local protein synthesis, receptor modification or insertion, membrane remodeling, and protein degradation (Achterberg et al., 2014; Hafner et al., 2019; Simmons et al., 2024).

In spike timing-dependent plasticity (STDP), the relative timing of presynaptic and postsynaptic spikes determines the directionality and magnitude of long-term change. In the rodent cortex, a presynaptic spike arriving 10 ms before a postsynaptic spike typically induces LTP, whereas if the order is reversed (postsynaptic before presynaptic), LTD is induced (Lu et al., 2007; Shin et al., 2006). However, these synaptic learning rules can be complex and are tuned by various factors including dendritic location, the presence of neuromodulators, developmental stage, and species (Froemke et al., 2005; Itami and Kimura, 2012; Seol et al., 2007).

The aforementioned types of plasticity are commonly summarized as associative plasticity that overall acts as a positive feedback mechanism. In contrast to associative plasticity, homeostatic plasticity operates on negative feedback mechanisms. It aims at keeping individual neuronal activity within a physiological range upon perturbations in network activity (Keck et al., 2013). Still, it remains an ongoing debate how the differential expression of associative and homeostatic synaptic plasticity is orchestrated. Previous studies identified commonalities in downstream pathways, such as retinoic acid-mediated control of AMPAR content at synaptic sites (Arendt et al., 2015; Lenz et al., 2021), the involvement of the spine apparatus organelle (Kruse et al., 2024; Speranza et al., 2022), or the recruitment of local protein synthesis near synaptic sites (Sun et al., 2021). A dose-dependent expression of these two forms of plasticity may arise from the recruitment of calcium-dependent signaling pathways with differential calcium affinity. Here, the low-affinity CaMKIV mediates synaptic downscaling upon persistent increases in network activity, whereas high-affinity CaMKII mediates LTP upon short-term increases in cytosolic Ca^2+^ (Ibata et al., 2008; Xiao et al., 2023). Moreover, molecular switches as recently described for the phosphorylation of Shank3, crucially determine the directionality of plasticity (Wu et al., 2022). Despite these significant advances, further studies are needed to improve our understanding of the regulation of plasticity.

Of note, fundamental synaptic mechanisms were mostly revealed from rodent and other animal studies. It warrants further investigation to what extent adult human cortical synapses obey the same rules or whether they have unique properties. In the following sections, we discuss how recent investigations have directly probed synaptic transmission and plasticity in human cortical tissue to specifically address this point.

## Investigating synaptic transmission in the human neocortex

The human brain is protected through the skin, skull, and meninges. Although indirect measurements of electrical brain activity (e.g., EEG) or biomarker analyses from peripheral blood are well established diagnostic procedures in clinical practice, direct conclusions on synaptic transmission and plasticity remain challenging. Numerous studies have examined cortical and synaptic architecture in post-mortem human brain tissue. While post-mortem tissue enables comparative (ultra)structural analyses across brain regions (Bothwell et al., 2001; Glausier et al., 2025; Montero-Crespo et al., 2021; Scaduto et al., 2020), functional studies are constrained by tissue degradation and variability in the post-mortem interval. Recent advances employ neurosurgical resections to assess neural circuits in the adult human cortex. Cortical resections are an essential part of different neurosurgical procedures, such as tumor resection or epilepsy surgery. These tissue samples can be used for basic research assessment upon rigorous ethical review. For the use of these tissue samples, several basic requirements need to be fulfilled: (1) Consent of patient for the use of tissue sample in basic research, (2) tissue resection is a mandatory part of the surgical procedure and does not relate to basic research questions, (3) the tissue sample is not needed for diagnostic purposes, and (4) tissue handling does not significantly prolong the surgical procedure.

**FIGURE 2:**
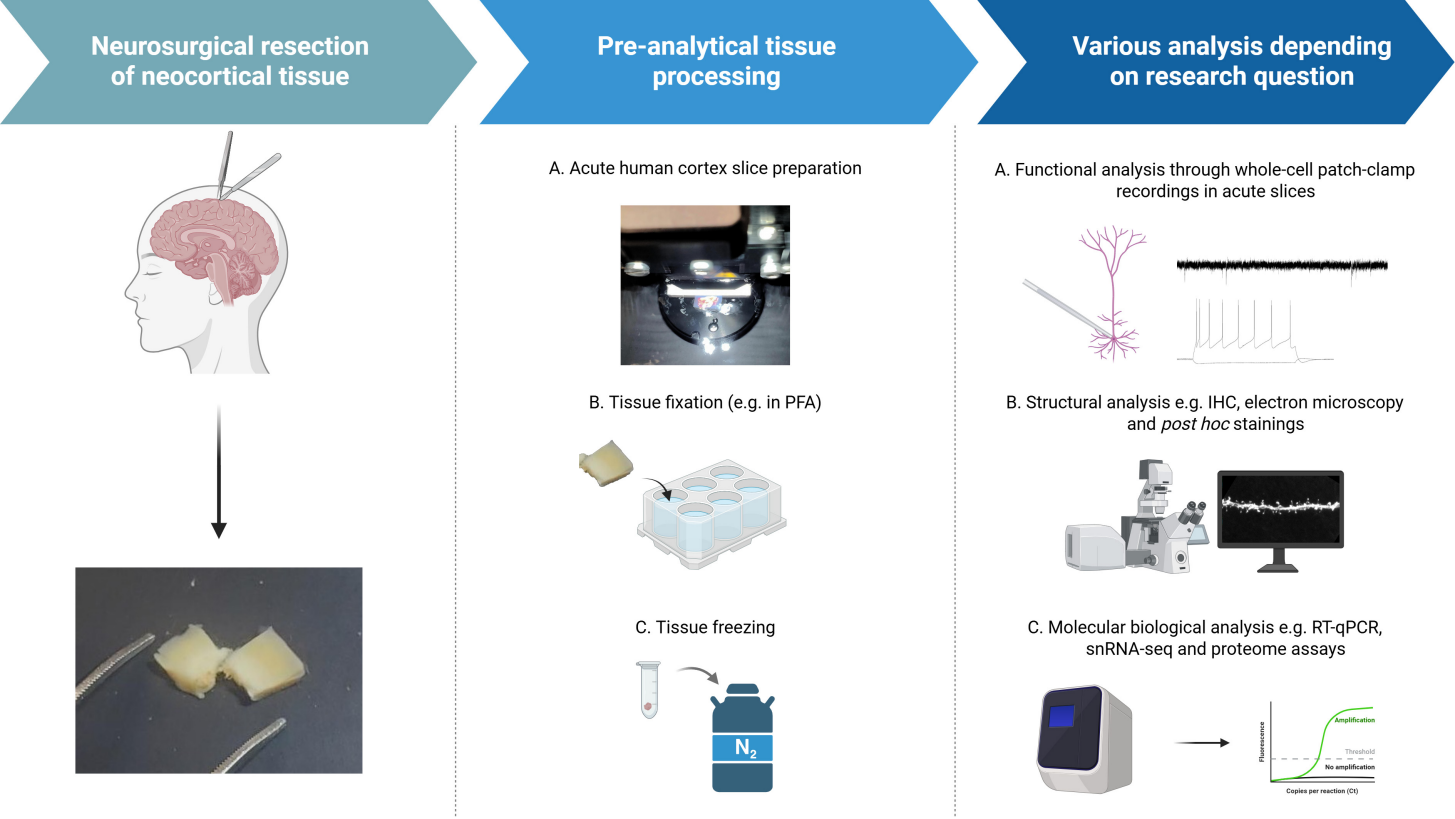
Workflow for experimental assessment of the human neocortex. Cortical sections of neurosurgical procedures can be used for experimental assessment. Tissue processing and analysis include acute slices for *ex vivo* functional characterization of cortical neurons, fixation and subsequent immunohistochemistry and freezing for different molecular biological analyses. Created with Biorender.

Recently, best practice guidelines for the neurosurgical resection of neocortical tissue for basic research questions have been implemented (Straehle et al., 2023). Tissue samples can be either fixed for microscopic analyses, frozen or dissociated for molecular biology assessment (e.g., proteome, snRNASeq), or further processed to obtain living brain slice preparations (Figure 2). These acute human cortical slices (∼400 μm thickness) are prepared along the pia-white matter-axis with a vibratome. When obtained under appropriate conditions, viable slices maintain their cortical lamination and cellular integrity providing suitable conditions for electrophysiological recordings similar to rodent slice preparations. Using whole-cell patch-clamp recordings, synaptic transmission and signal integration along dendritic trees can be assessed with subcellular resolution (Figure 2). Using *post hoc* visualization of cells, functional and structural properties, such as dendritic complexity and morphological features of dendritic spines, can be correlated. Further developments established multi-neuron patch-clamp recordings, which allow a mapping of single-unit connectivity and dynamics in the human neocortex (Peng et al., 2019). Using direct comparisons to the mouse neocortex, species-related differences can be revealed. Overall, investigating synaptic transmission in the human neocortex therefore requires an integration of methodologies: acute slice physiology for functional characterization, anatomical reconstructions (light and electron microscopy) for structural context, and molecular profiling for the investigation of underlying mechanisms (Figure 2).

Although these methodologies are well-established, recordings in human tissue remain rare. Due to the heterogeneity of patients, including age, sex, and medical record, a substantial inter-individual variability must be considered in experimental design and data interpretation. Regional differences in cortical lamination and cell types might critically shape synaptic features. In addition, variability in transportation time and tissue sample sizes might represent significant confounding factors. Moreover, for the investigation of synaptic transmission under physiological conditions, it is important to perform experiments with access tissue that is not overtly affected by the pathology (e.g., brain tumor).

## Specialization of the human neocortex – glutamatergic cell types

The cell composition of the human and mouse neocortex is highly different (Fang et al., 2022). A lower proportion of neurons and a higher proportion of glial cells can be found in human neocortical regions when compared to their orthologs in mice. In addition, the proportion of interneurons to the overall neuronal population is higher in humans, indicating a strong inhibitory tone in the human neocortex. Moreover, a dominance from intratelencephalic-projecting (IT) neurons over extratelencephalic-projecting (ET) neurons is evident in the human brain, which aligns with evolutionary supragranular expansion.

Supragranular expansion is a defining evolutionary feature of the human neocortex. Expansion of the human neocortex is accompanied by a functional, structural, and molecular diversification of neurons. The structural and functional specialization of cell types within the human supragranular neocortex highlights critical evolutionary divergences from rodent models, reflecting distinct computational capacities. Recent advances in single-nuclei RNA sequencing (snRNASeq) and integrated electrophysiological and morphological techniques have substantially broadened our understanding of neuronal diversity within these layers.

Specifically, an increased diversity of supragranular glutamatergic IT neurons was reported, compared to the mouse brain. Human L2/3 neurons match transcriptomic profiles also found in the mouse brain, while additional glutamatergic neuron types were found in deep L3 (Hodge et al., 2019). Berg et al. (2021) demonstrated that layers 2 and 3 of the human middle temporal gyrus feature five distinct glutamatergic neuronal transcriptomic types – LTK, GLP2R, FREM3, CARM1P1, and COL22A1 – whereas the equivalent layers in mice typically show only three types. Specific for the human brain, deep L3 glutamatergic cell types form long-range projections that are specifically vulnerable to degeneration in Alzheimer’s disease (Berg et al., 2021). In general, molecularly-defined glutamatergic cells show higher diversity in humans and are less layer-restricted. The morpho-electric characteristics of recorded cells in both human and mouse neocortex reflect transcriptomic diversity.

**FIGURE 3:**
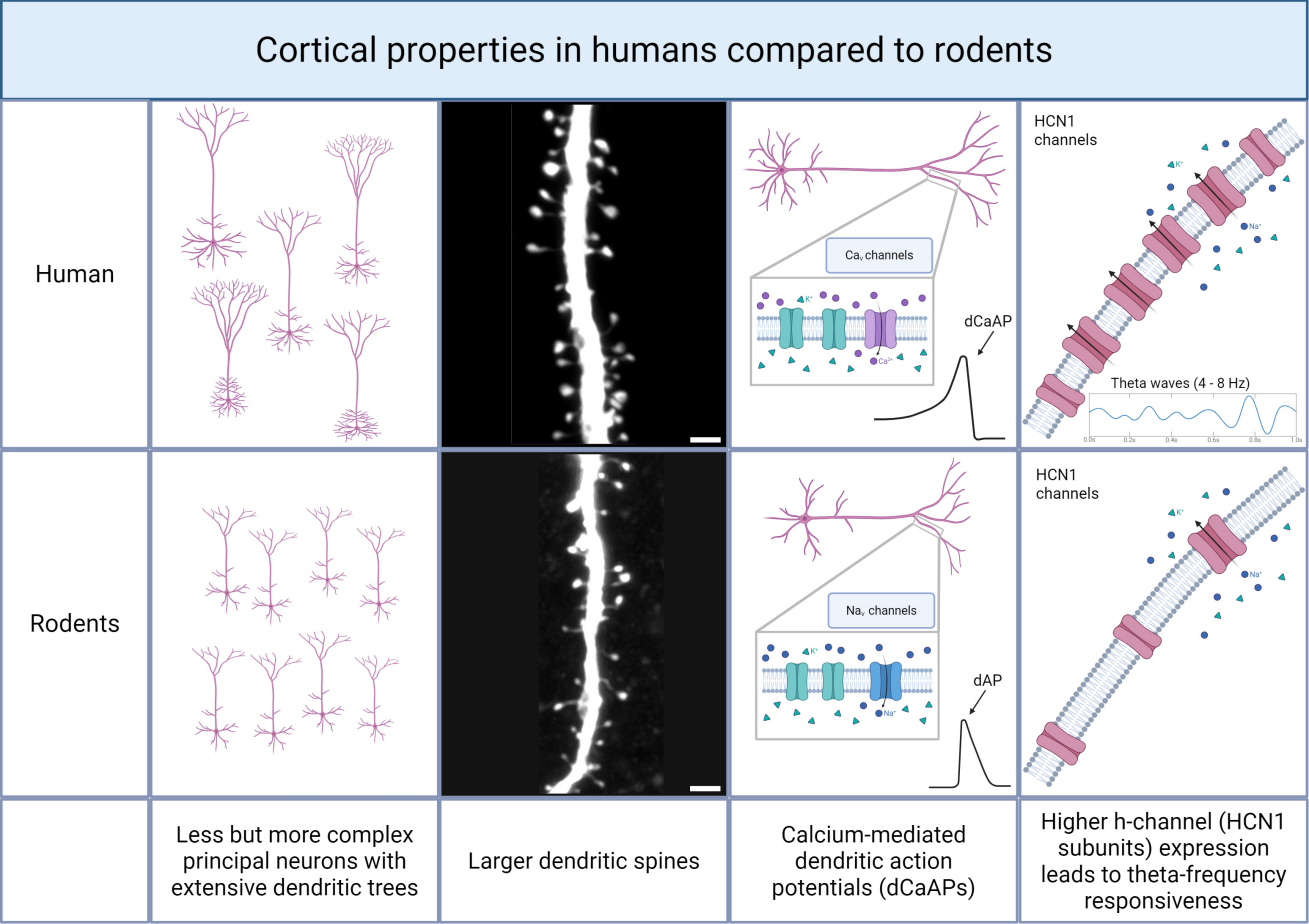
Differences between the human and the rodent neocortex. Cortical properties show differences between humans and rodents. In the human neocortex, less principal neurons are present, but those show a higher dendritic complexity (1^st^ panel). Additionally, human pyramidal neurons have larger dendritic spines (2^nd^ panel; scalebar 2 μm). Moreover, dendrites in the human neocortex show calcium-mediated action potentials, whereas in the rodent cortex, dendritic action potentials are mainly mediated by sodium channels (3^rd^ panel). Principal neurons in the human neocortex show a theta-rhythm preference, which is based on a high expression and open probability of HCN channels (4^th^ panel). Created with Biorender.

The density of neurons is higher in the mouse brain compared to the human brain. At the same time, the density of neurons shows a depth gradient in the human brain, with highest densities in L2, whereas equal densities across layers were reported for the mouse brain (Berg et al., 2021). Morphologically, human neurons within layers 2 and 3 exhibit significantly greater variability and complexity than rodent counterparts (Figure 3). The size of neuronal somata in humans doubles from superficial to deeper parts of layer 3, accompanied by substantial heterogeneity in dendritic morphology. Notably, dendrites in human neurons extend significantly further and have a more complex branching, supporting higher-order integrative processing capabilities (Hodge et al., 2019). Recent work specifically revealed much higher branching density in perisomatic regions, i.e., basal and oblique dendrites in human pyramidal neurons (Kanari et al., 2025). Additionally, human layer 2/3 pyramidal neurons frequently have larger dendritic spines than those found in rodents, enabling improved synaptic integration (Benavides-Piccione et al., 2025).

Functionally, multipatch electrophysiological studies have identified distinct patterns of connectivity and excitability between human and mouse cortical neurons. Recent findings suggested that human pyramidal neurons exhibit sparse but robust recurrent excitatory connectivity within supragranular layers, contrasting the more uniformly distributed connectivity seen in mice (Seeman et al., 2018). Moreover, human cortical neurons exhibit calcium-mediated dendritic action potentials (dCaAPs; (Gidon et al., 2020)), whereas dendritic action potentials in the rodent cortex are mainly sodium-mediated (Bast et al., 2025). This reflects a heightened dendritic excitability in human cortical neurons that enables complex computational functions. The evolution of neurons in supragranular layers of the human neocortex therefore aligns with functional specialization that corresponds to higher brain function.

Morphological and functional specialization is underlined by species-specific genetic programs that affect both neuronal diversity and the laminar distribution pattern. Through multiplexed error-robust fluorescence *in situ* hybridization (MERFISH), previous work demonstrated that human cortical cells have specialized patterns of intercellular proximity and interaction not observed in mice, significantly influencing local circuit dynamics and signaling pathways (Fang et al., 2022). Here, interactions between neuronal and non-neuronal cells, e.g., astrocytes and microglia, are enriched in the human cortex, implying specialized mechanisms for maintaining synaptic homeostasis and modulating synaptic plasticity. Specifically, a higher number of perineuronal oligodendrocytes was found in the human brain, which might indicate higher energy demands. Moreover, a preferential enrichment of microglia-to-excitatory neuron contact sites was evident, indicating functional interactions between microglia and neurons.

Intrinsic electrophysiological specialization also contributes significantly to the function of human cortical neurons. An increased expression and functional contribution of h-channels (HCN1 subunits) in human supragranular pyramidal neurons, unlike in mice, leads to pronounced subthreshold membrane properties and theta-frequency responsiveness (Figure 3; Kalmbach et al., 2018). Since theta-band rhythms are associated with attention and memory, this frequency preference might serve higher cognitive function in humans (Rutishauser et al., 2010). These findings were recently supported by reports demonstrating considerable electrophysiological diversity in human pyramidal neurons characterized by distinctive sag currents, which further contribute to the unique computational abilities of human cortical microcircuits (Moradi Chameh et al., 2021). These properties of the human neocortex were recently discussed by de Kock and Feldmeyer (2023). Although unitary excitatory postsynaptic potentials (uEPSPs) remain relatively conserved, substantial divergence in anatomical and functional properties of neurons might cause differential processing of synaptic inputs in supragranular layers across species.

## Specialization of the human neocortex – excitatory synaptic transmission

Estimates based on computational modeling suggested that a single human L2/3 pyramidal neuron integrates input from approximately 20,000–30,000 excitatory synapses, roughly three-fold higher than the ∼10,000 synapses typical for a rodent L2/3 pyramidal neuron (Eyal et al., 2018). Notably, spines on human cortical neurons are larger in volume and longer than rodent spines, implying a larger postsynaptic density and potentially more postsynaptic receptors per synapse (Figure 3; Benavides-Piccione et al., 2025). However, within-subject variability of spine sizes depending on the analyzed cortical region, such as the occurrence of larger spines in association cortical areas, needs to be considered.

Synaptic transmission in the human cortex has been extensively investigated during recent efforts, which led to the establishment of open data platforms (Campagnola et al., 2022). These data revealed that excitatory synaptic dynamics align with postsynaptic cell subclasses, whereas the properties of inhibitory synapses align with the presynaptic subclass. In addition to multiple commonalities between the mouse and human brain, differences are particularly evident in excitatory-to-excitatory synaptic dynamics in supragranular layers (Peng et al., 2024). Here, a substantial reliability of synaptic connections exists in the human brain with widely depressing dynamics and very fast recoveries. In contrast, rodent L2/3 excitatory synapses onto pyramidal cells show both facilitation or depression with slower recovery (Campagnola et al., 2022). Stronger and more reliable synaptic communication than those found in mice is characterized by approximately threefold greater amplitudes of excitatory postsynaptic potential (EPSP) and virtually no failure rates (Hunt et al., 2023). The enhanced human synaptic strength arises primarily from significantly larger AMPAR- and NMDAR-conductance per synapse, leading to prolonged EPSP durations. Notably, synaptic properties depend on depth across layer 2 and 3 (Campagnola et al., 2022).

Computational modeling summarized the properties of excitatory synapses in supragranular pyramidal neurons (Eyal et al., 2018). While connections are more abundant in the human than in the mouse brain, larger somatic unitary EPSPs (∼0.3 mV) are found in human supragranular pyramidal neurons compared to mouse (∼0.1–0.2 mV). The models predict particularly large AMPAR- and NMDAR-conductance per synaptic contact (0.88 and 1.31 nS, respectively) and a steep dependence of the NMDAR-conductance on voltage. The estimated magnitude of EPSPs at the spine head (12.7 ± 4.6 mV), spine base (9.7 ± 5.0 mV), and soma (0.3 ± 0.1 mV) demonstrate a considerable attenuation of signals between the postsynaptic density and the cell soma. Furthermore, matching the shape and firing pattern of somatic Na^+^-spikes provided estimates for the density of the somatic/axonal excitable membrane ion channels, which led to the prediction that 134 ± 28 simultaneously activated synapses between pyramidal neurons are required for generating a somatic Na^+^-spike. Dendritic NMDAR-spikes, however, were triggered in the model when 20 ± 10 excitatory spinous synapses are simultaneously activated on individual dendritic branches. These computations indicate that the number of synchronous inputs to reach the spike threshold is comparable in mice and humans. Given the larger number of synapses, this finding indicates increased signal integration properties in human neurons, which coincides with the evolutionary network expansion.

Dendritic spines are the predominant structural correlate of excitatory synapses in the cerebral cortex and, accordingly, critically shape cortical computation and network performance. Their structure is tightly linked to function. Recent high-resolution analyses demonstrate distinct characteristics of human dendritic spines compared to the mouse neocortex (Benavides-Piccione et al., 2025). Systematic electron-microscopic comparisons across cortical layers in human, rat, and mouse indicate higher synaptic densities in rodents. Notably, the difference is most prominent in layers I and IV, where synaptic density in mouse cortex is nearly threefold higher than in human cortex (DeFelipe et al., 2002). However, comparative studies remain challenging due to uncertainties in defining regional homologies across species. Focusing on hippocampal CA1 pyramidal neurons, which is an evolutionarily conserved archicortical region, human tissue shows lower spine density but larger spine volume and greater spine length than mouse tissue, recapitulating neocortical differences (Benavides-Piccione et al., 2025). Within the human brain, regional variation is evident: the hippocampus exhibits higher spine density with smaller, shorter spines than the temporal or cingulate cortex. Moreover, sex-related differences have also been reported, with higher synaptic densities in male cortical samples (Alonso-Nanclares et al., 2008). In recent years, *in vivo* PET imaging of synaptic density has become feasible using a tracer targeting synaptic vesicle glycoprotein 2A [SV2A; (Finnema et al., 2016)]. This approach enables quantitative comparisons in living humans across neurological and psychiatric conditions and can be cross-validated against surgical access tissue or post-mortem samples. Together, species- and region-specific data underscore a specialization of the human cortex, while general scaling relationships between spine size and density appear conserved across species.

Moreover, volume electron microscopy has revealed fundamental ultrastructural features of synaptic organization in the human neocortex, including layer-specific distributions of symmetric and asymmetric synapses in the medial entorhinal cortex (Domínguez-Álvaro et al., 2021; Plaza-Alonso et al., 2025), primary cortical areas (Cano-Astorga et al., 2024), anterior cingulate and temporopolar cortex (Cano-Astorga et al., 2023), and temporal neocortex (Cano-Astorga et al., 2021). High-resolution tomographic approaches and 3D reconstructions have characterized active zones and synaptic vesicle pools across layers. In layer 6, substantial heterogeneity of synaptic contacts has been identified, including large active zones and sizeable readily releasable pools, consistent with high reliability and broad functional repertoires (Schmuhl-Giesen et al., 2022). In line with this, layer 5 excitatory boutons in the human temporal neocortex display large presynaptic active zones, prominent postsynaptic densities, and expanded vesicle pools that exceed those reported in other species (Yakoubi et al., 2019). Of note, the basic tripartite synapse architecture appears preserved across species and neocortical layers. Many studies have focused on deeper layers of the human neocortex or hippocampus and provide important insights into the (ultra)structural specialization of the human brain. Quantitative data on synaptic organization enable mechanistic explanations and even predictions of network and microcircuit function. Finally, they support computational simulations of parameters governing synaptic transmission and plasticity. This is of particular importance because such modeling helps to overcome experimental constraints inherent to human tissue work. Extending these quantitative datasets to the evolutionarily expanded supragranular layers will substantially advance our understanding of computations that may be unique to the human cortex.

Using petavoxel-scale electron microscopy, a recent study reconstructed a 1 mm^3^ volume of human temporal cortex and revealed novel ultrastructural characteristics (Shapson-Coe et al., 2024). In this volume, approximately 150 million synapses (111 million excitatory and 39 million inhibitory) across ∼57,000 cells were identified. Excitatory synapses are most densely localized in layers 1 and 3, while inhibitory contacts peak in layer 1. Pyramidal neuron compartments (spines vs. somata/AIS) show conserved excitatory–inhibitory spatial patterns, mirroring those observed in mouse cortex. These findings underline that many fundamental synaptic features, such as compartmentalized excitatory/inhibitory targeting and predominance of one synapse per axon-to-dendrite pair, align closely with mouse neocortex. While thousands of mostly weak synaptic contacts were identified, rare but strong connections emerged in the human cortex where single axons formed up to 50 synapses onto the same postsynaptic neuron. The discovery of dense, multi-synaptic strong links and the considerable volume of synapses suggests that while the basic rules are conserved, humans possess unique synaptic architectures that may enhance computational efficacy and network integration.

Human cortical dendritic spines exhibit further notable specialization. Spine density varied by sex and dendritic compartment with apical dendrites showing higher densities (Schünemann et al., 2025). However, synaptic features can be influenced by various endogenous and exogenous factors in patients. We previously reported an age-dependent decline in dendritic spine densities, while excitatory neurotransmission on the functional level remains stable (Lenz et al., 2024). However, other reports suggested that the synaptic NMDA/AMPA ratio decreases during aging, which is caused by a reduced recruitment of GluN2B containing NMDA-receptors in older individuals (Pegasiou et al., 2020). This coincides with age-dependent changes observed in inhibitory circuitries, as demonstrated for weakening of action potentials in fast-spiking interneurons (Szegedi et al., 2024). In addition, dendritic pathology, spine loss and synaptic reorganization can be found in both neocortex and hippocampus from epilepsy patients (Bod et al., 2023; Rossini et al., 2021). While in type II cortical dysplasia dendritic complexity might be increased, a consistent reduction in dendritic spines with filopodia emerging from the soma is evident in epileptic cortices. Outside type II dysplastic regions, no alterations of dendritic and spine morphologies can be found.

## Specialization of the human neocortex – excitatory synaptic plasticity

**FIGURE 4:**
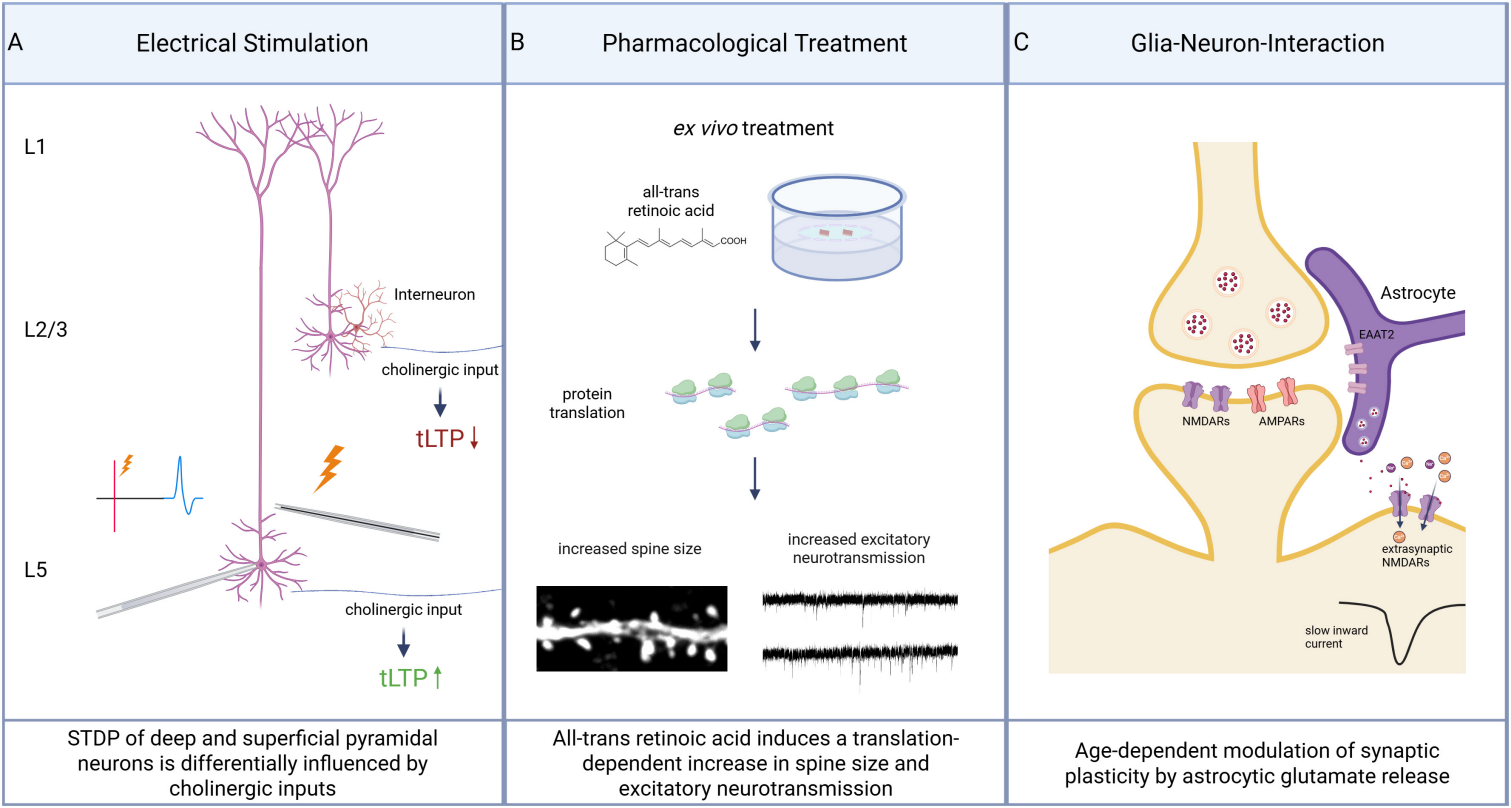
Examples of different mechanisms of synaptic plasticity in the human neocortex. (A) Spike-time dependent plasticity (STDP) can be induced by coordinated pre- and postsynaptic stimulation and induces timing-dependent long-term potentiation (tLTP). Cholinergic input differentially regulates tLTP induction. At superficial (L2/3) pyramidal neurons it leads to a reduction of tLTP expression via interneurons. At deep (L5) pyramidal neurons cholinergic input strengthens tLTP by directly targeting pyramidal neurons. (B) Pharmacological treatment *ex vivo* with all-trans retinoic acid induces a strengthening of excitatory neurotransmission in human neocortical pyramidal neurons. This is reflected by an increase in dendritic spine volume and changes in spontaneous excitatory postsynaptic currents. Mechanistically, these effects depend on protein translation. (C) Astrocytes modulate synaptic plasticity via glutamate reuptake and release. Via glutamate-dependent activation of extrasynaptic GluN2B-containing NMDA receptors (NMDARs) slow inward currents (SICs) are generated in neocortical pyramidal neurons. These inward currents have been shown to be age-dependent: while neurons of young patients show pronounced SICs with timing-dependent synaptic facilitation, neurons of elderly patients do not depict SICs. Created with Biorender.

While most plasticity mechanisms were delineated in animal and cell culture models, it has become clear that the human brain also supports robust forms of synaptic plasticity (Beck et al., 2000). These early studies using resected human hippocampal tissue demonstrated that classical NMDA receptor-dependent long-term potentiation (LTP) can be reliably induced at synapses (e.g., in the perforant path – dentate gyrus circuit), indicating that activity-dependent synaptic strengthening exists in the human CNS. Recent research, however, revealed unique specializations of excitatory synaptic plasticity in the human neocortex that distinguish it from other species (Figure 4).

Excitatory synaptic plasticity in the human neocortex is regulated by cholinergic inputs from the basal forebrain with substantial differences in superficial and deep layers (Verhoog et al., 2016). Recent experiments in human and mouse prefrontal cortex showed that activating nAChRs can have opposite effects on STDP depending on the respective layer. In deep layers, endogenous acetylcholine release or nicotine activation facilitates long-term potentiation of glutamatergic synapses by acting on postsynaptic nAChRs located on dendrites of pyramidal neurons. In contrast, in superficial layers, cholinergic stimulation tends to suppress LTP, which is mediated indirectly via nAChRs on GABAergic interneurons that enhance inhibition onto pyramidal neurons. This layer-specific cholinergic control of plasticity is conserved from rodents to humans and illustrates a potential mechanism by which the basal forebrain can fine-tune cortical information processing. Depending on acetylcholine levels, which are influenced by behavioral or cognitive states, certain cortical layers may be gated to undergo synaptic strengthening while others are stabilized, thereby optimizing learning and network function in a context-dependent manner.

A key molecule in the regulation of synaptic plasticity is the vitamin A derivative all-trans retinoic acid, which was identified as a regulator of excitatory synaptic strength in both associative and homeostatic plasticity (Hsu et al., 2019; Thapliyal et al., 2022). In adult human cortical neurons, all-trans retinoic acid (atRA) can acutely induce synaptic plasticity even in *ex vivo* brain slices (Lenz et al., 2021). Application of atRA to human neocortical tissue was found to enlarge dendritic spines and strengthen excitatory synaptic transmission, reflecting coordinated structural and functional potentiation of synapses (Figure 4). Moreover, the plasticity associated spine apparatus organelle represents a target for atRA in the human brain. The atRA-induced potentiation depends on protein synthesis supporting the idea that endogenous RA signaling might contribute to experience-dependent synaptic changes in the human cortex. Notably, the relevance of atRA-induced synaptic plasticity in the human brain indicates novel therapeutic perspectives to ameliorate synaptic or cognitive deficits observed in disorders like depression. Moreover, atRA is involved in regulating the development, molecular patterning, thalamocortical connectivity and dendritic spinogenesis in the human prefrontal cortex (Shibata et al., 2021). By integrating analyses of human tissue and animal models, this recent study demonstrated that atRA is required for proper expression of regional gene programs in the prenatal frontal cortex, leading to physiological dendritic spine formation and establishing long-range thalamocortical connections. These findings indicate that atRA sets the stage for plasticity of the human neocortex by ensuring the cortex is properly wired and laminated.

Plasticity can be strongly influenced by neuromodulators that signal metabolic states and arousal. Adenosine, for instance, is an inhibitory neuromodulator in the brain that tonically suppresses excitatory synaptic transmission and neuronal excitability via A1 adenosine receptors (A1R). Caffeine, which is an inhibitor of the A1R, increases pyramidal neuron excitability and excitatory synaptic transmission (Kerkhofs et al., 2017). It elicits these effects by blocking A1R-mediated inhibition, which effectively disinhibits the normal suppressive action that endogenous adenosine has on synapses. Specifically, caffeine restores the excitability and synaptic strength that adenosine would otherwise reduce, particularly by antagonizing postsynaptic A1Rs on pyramidal neurons. Interestingly, the endogenous adenosine tone in the human cortex has a stronger effect at synapses than on the neuron’s intrinsic excitability, suggesting that A1Rs at synaptic sites are highly sensitive. Consistent with that, caffeine shows a distinct preference for blocking synaptic A1Rs over extrasynaptic ones, likely due to different receptor micro-environments (Kerkhofs et al., 2017).

Finally, emerging evidence pointed to a critical role of non-neuronal cells in human synaptic plasticity (Figure 4). Astrocytes actively participate in modulating synapses by reuptake and release of glutamate and other gliotransmitters. In both mice and humans, astrocyte-driven glutamate release can evoke slow inward currents (SICs) in nearby neurons through activation of extrasynaptic NMDA-receptors. These SICs induce synaptic plasticity in both mice and humans (Csemer et al., 2023). Importantly, this phenomenon is age-dependent. In cortical slices from young mice and humans, SICs occur frequently and contribute to plasticity maintenance. However, the occurrence of SICs declines with aging – in adult human cortical samples, SIC events become infrequent and disappear beyond a certain age threshold. The loss of these astrocyte-mediated NMDAR currents in older tissue correlates with impairments in synaptic plasticity that develop with age. Thus, a close astrocyte–neuron interaction appears to orchestrate excitatory synaptic plasticity in the young brain, and dysfunctional astrocytes in the aging brain may contribute to the decline in plasticity and cognitive flexibility observed in older adults.

Overall, these studies highlight that while core mechanisms of synaptic plasticity are preserved in the human neocortex, there are important specializations.

## Inhibitory cells and synapses in the human cortex

The role of inhibition is inseparable from cortical circuit function. Detailed investigations revealed a crucial role for inhibition in sculpting the timing and gain of excitatory synapses and cellular excitability. A pivotal role has been identified for inhibitory neuronal networks in cortical rhythm generation, such as sharp-wave ripples (SWRs), theta-, and gamma oscillations reflecting differential cognitive states (Gan et al., 2017; Pastoll et al., 2013). Interneurons are a diverse cell population with distinct functional and structural properties, which form inhibitory synapses on both excitatory and inhibitory neurons. The compartment- and layer-specificity of their axonal connections has an impact on information processing and cognitive function, as demonstrated for the role of dendritic inhibition on fear learning (Lovett-Barron et al., 2014). Consequently, malfunction of the inhibitory system promotes pathological network states, such as epilepsy (Tóth et al., 2021).

The human cortex has a 2.5-fold higher number of interneurons compared to the mouse brain (Loomba et al., 2022). An expansion of interneurons that target other interneurons has been observed while the wiring pattern between interneurons and pyramidal neurons shows similarities across species. However, a strong body of evidence suggests the functional and structural specialization of the inhibitory system in the human brain. Cortical fast-spiking interneurons show increased excitatory synapse strength, reduced dendritic complexity, larger dendrite diameter, faster AP initiation, and tuned inhibitory output facilitation mediating a fast inhibition of pyramidal neurons in the human brain (Wilbers et al., 2023). Notably, inhibitory autapses – self-innervating synaptic connections in GABAergic interneurons – are prevalent in both mouse and human brains. Here, autapses on parvalbumin-expressing basket cells in supragranular layers inhibit repetitive firing and thereby regulate the timing of basket cell discharges (Szegedi et al., 2020).

Further specialization of inhibition in the human brain has been revealed by describing unique inhibitory neurons that target pyramidal cells. The rosehip neuron of layer 1 forms inhibitory synapses onto the distal apical dendrites of layer 2/3 pyramidal neurons (Boldog et al., 2018). These GABAergic synapses are positioned to selectively modulate dendritic excitability and signal propagation. Specifically, rosehip inputs can veto or dampen back-propagating action potentials and dendritic calcium spikes in the tuft, without directly inhibiting the perisomatic region. This spatial specificity, which is distal dendritic inhibition, resembles the actions of neurogliaform cells in the rodent brain with a unique molecular signature that defines the human rosehip neuron. Functionally, extending inhibitory control in the human cortex for promoting nonlinear input integration in distal dendrites can potentially affect plasticity induction, since dendritic spikes often trigger NMDAR-activation and plasticity cascades (Brandalise et al., 2016). In the broader sense, it highlights that inhibitory synapses in humans are not only more abundant but also more diversified.

Moreover, the strength and reliability of synaptic connections within inhibitory circuitries of the human neocortex are distinct from the rodent brain (Molnár et al., 2016). As a common network pattern, fast-spiking parvalbumin (PV) interneurons in both humans and rodents receive strong synaptic inputs from pyramidal cells. The discovery of multi-vesicular release at human pyramidal cell → PV synapses suggested that each such synapse can elicit stronger depolarization per spike than in rodents. Feed-forward and feedback inhibitory signals, which are mediated by PV interneurons on perisomatic pyramidal cell compartments, are therefore particularly strong in human circuits. These findings align with the observation of complex events in neocortical circuits that are triggered by solitary action potentials in layer 2/3 pyramidal neurons (Szegedi et al., 2016, 2017). Consequently, pyramidal cell firing can strongly recruit inhibition onto its neighbors via PV cells. This might crucially contribute to oscillatory synchrony, specifically at higher frequency bands. In line with the high reliability of this synapse, pyramidal cell projections to PV interneurons typically show depressing synaptic characteristics (Kim et al., 2023). Other synapses onto interneurons broadly show facilitating dynamics. Given the postsynaptic specialization for compartmental innervation in cortical networks, i.e., perisomatic and dendritic inhibition, these differential synaptic dynamics crucially shape cellular computations.

Inhibitory neurotransmission can be modulated under various physiological and pathological conditions. Thus, several studies investigated whether neuromodulation of inhibition, e.g., through acetylcholine, might be regulated differently in the mouse and human cortex. Although the presence of cholinergic neuromodulation is conserved from mice to humans, previous work demonstrates layer- and cell-type-specific differences of cholinergic modulation between mouse and human cortical circuits with an impact on synaptic plasticity (Verhoog et al., 2016). Cholinergic projections can target both pyramidal cells and interneurons. Specifically, a strong nicotinic recruitment of layer 1 interneurons was previously reported and a broad range of shared properties of layer 1 interneurons was found in mice and humans (Poorthuis et al., 2018). In addition to physiological commonalities, human layer 1 interneurons show distinct specializations, such as higher input resistance and lower action potential threshold. Further modulating effects were described for the glutamatergic system through activation of group III mGluRs in dendritic spine targeting interneurons (Lukacs et al., 2023). While activating group III mGluRs facilitates spontaneous inhibitory postsynaptic currents (sIPSCs) in calbindin-positive double bouquet cells, depression of sIPSCs occurs in parvalbumin-expressing dendrite-targeting cells. Thus, glutamate can differentially regulate GABAergic transmission in distinct cortical cell types, which target excitatory inputs onto pyramidal neurons.

## Discussion and perspectives

The use of human brain resections provides the unique opportunity to study membrane properties, synaptic transmission and regional specialization in an intact tissue organization. Although basic principles of membrane physiology, synaptic transmission, and information coding are conserved throughout evolution and research models, a specialization of the human cortex is evident (de Kock and Feldmeyer, 2023). For example, adult human synapses can recover faster from short-term depression than rodent synapses, enabling higher-frequency information transfer and efficient communication between neurons. This high-bandwidth synaptic communication is thought to underlie the complex computational capacity of human cortical circuits. Studying living human cortical slices thus bridges the gap between animal models and clinical neuroscience, ensuring that uniquely human aspects of synaptic function are revealed. It is therefore essential to further expand the sustainable use of human neocortical material in the translational assessment of synaptic transmission, plasticity, and network dynamics.

A major challenge in the employment of human brain tissue comes from the heterogeneity of patients, cortical resection sites, and the cellular diversity, which is particularly prominent in supragranular layers due to evolutionary expansion. Previous work revealed that biographic metrics significantly influence the structure of cortical networks, as demonstrated for age-dependent changes in dendritic spines (Lenz et al., 2024). It warrants further investigation whether medication, lifestyle, environmental factors, and the socioeconomic background also shape synaptic transmission. Of note, previous studies established a firm link between intelligence and pyramidal neuron structure (Goriounova et al., 2018; Heyer et al., 2022). Individuals with higher IQ scores tend to have larger, more complex dendrites in their cortical pyramidal cells, as well as thicker cortical layers with lower neuron density and faster action potential kinetics in those neurons. These features are thought to enable more efficient information processing in neuronal networks, suggesting that cognitive ability is reflected by neuronal architecture. Nevertheless, even under best-practice conditions, the degree of experimental standardization achievable with human brain samples remains limited. Rather than being a drawback, this inter-individual variability can be informative, since the experimental outcome identifies which neurobiological principles are invariant and which differ from person to person. In the long term, insights gained from such variability may improve clinical translation by informing personalized approaches and highlighting population-level vs. individual-specific targets in neurological therapy.

Regional specialization is a fundamental neuroanatomical principle of the mammalian brain. As recognized by Korbinian Brodmann, specialization is reflected by differences in cortical lamination, giving rise to “granular” (well-defined layer IV) and “agranular” (lacking layer IV) cortex architectures. Beyond differential lamination, recent findings on the diversification of glutamatergic neurons in supragranular layers add another level of complexity (Berg et al., 2021). As a consequence, the composition and wiring of cortical columns, which are considered the functional units of the human neocortex, are highly variable. The systematic assessment of cellular connectivity and transmission rules in human micronetworks is therefore a crucial component in future research (Peng et al., 2019). While substantial work investigated neocortical resections in the past years, insights in other human brain regions remain limited. Expanding research to resections of the hippocampus (Mertens et al., 2024) or the cerebellum (Busch and Hansel, 2025) could be highly informative in this context. Notably, the complexity of human cerebellar Purkinje cells is considerably high. It is therefore interesting to speculate that the computational capacitance of human cerebellar Purkinje cells outperforms their counterparts in the mouse cerebellum.

The increasing size and complexity of the mammalian brain during evolution is accompanied by a corresponding increase in the size and morphological complexity of individual neurons. Human pyramidal neurons extend very long dendrites with thousands of spines, raising the questions of (1) how synaptic inputs on those distant dendrites can influence the soma effectively and (2) fast synaptic adjustments can be implemented at long distances to the soma. Dendritic complexity and increased cable lengths require the tuning of those processes that ensure synaptic autonomy and the efficient transmission of signals from distant synapses to the soma, since computational modeling indicates a substantial attenuation of electric signals from postsynaptic dendritic spines to the soma > 90% (Araya et al., 2006). Therefore, with increasing distance to the soma, the contribution of individual spines and dendritic segments to somatic potentials needs to be critically evaluated. Interestingly, recent evidence suggests that human neurons might offset some of these biophysical challenges through unique adaptations (Eyal et al., 2016). Beyond the biological relevance, this phenomenon additionally contributes to space-clamping effects when investigating synaptic transmission by somatic whole-cell patch-clamp recordings. Here, distant dendrites could undergo local nonlinear events like NMDAR spikes that are only partially reflected at the soma. For data interpretation, impactful space clamping effects must therefore be considered in complex human neurons. Moreover, unraveling the compartmental origin of synaptic potentials, such as apical or basal dendrites in pyramidal neurons, might provide important insights into information processing, which can be assessed by paired-recordings or uncaging experiments.

Given their cellular complexity, it can be assumed that human neurons substantially rely on local synaptic resources to maintain efficient communication (Bergmann et al., 2025). Local protein homeostasis (Sun et al., 2023), mitochondrial energy supply, and the regulation of synaptic calcium levels (Verma et al., 2022) are well established prerequisites for synaptic functionality in animal and cell culture models. Future studies will need to investigate how these local synaptic resources contribute to various forms of synaptic plasticity in the human brain both under physiological conditions, such as development and learning, and in disease states. Understanding the spatio-temporal regulation of synaptic resources in human neurons holds high innovative potential, as it could identify targets for novel therapeutic approaches, such as gene therapy.
